# Efficacy of previous failure on subsequent procedural outcomes of chronic total occlusion percutaneous coronary intervention: A systematic review and meta-analysis

**DOI:** 10.12669/pjms.42.6.16250

**Published:** 2026-06

**Authors:** Weiqiang Sun, Ailing Wu, Can Luo

**Affiliations:** 1 Weiqiang Sun, Department of Cardiovascular, Changxing County People’s Hospital, Huzhou, Zhejiang Province 313100, P.R. China; 2 Ailing Wu, Department of Electrocardiogram Room, Changxing County People’s Hospital, Huzhou, Zhejiang Province 313100, P.R. China; 3 Can Luo, Department of Cardiovascular, Changxing County People’s Hospital, Huzhou, Zhejiang Province 313100, P.R. China

**Keywords:** Chronic total occlusion, Coronary artery disease, Percutaneous coronary intervention, Complications

## Abstract

**Objective:**

The current study aimed to review evidence on the effects of prior failed percutaneous coronary intervention (PCI) for chronic total occlusion (CTO) on subsequent reattempted procedures.

**Methodology:**

Studies published on the databases of PubMed, Embase, Scopus, and Web of Science comparing outcomes of prior failed CTO-PCI with first-attempt CTO-PCI were included. Random-effects meta-analysis was conducted for baseline characteristics, success rates, and complications.

**Results:**

Six studies were included with 15,803 patients of which 3248 (20.6%) had prior failed PCI for CTO. Patients with prior failed CTO-PCI had significantly lower age, higher Japanese-CTO scores, higher incidence of tortuous vessels, increased contrast volume, and higher fluoroscopy time. The retrograde approach was more frequently used in prior failed cases. Meta-analysis showed that procedural (OR: 0.50 95% CI: 0.32, 0.77 I^2^=89%) and technical success (OR: 0.69 95% CI: 0.53, 0.88 I^2^=68%) was significantly lower in cases with prior failed CTO-PCI as compared to those undergoing first attempt CTO-PCI. There was no difference in the risk of major adverse cardiac events (OR: 1.14 95% CI: 0.62, 2.09 I^2^=47%) and individual complications between the two groups.

**Conclusions:**

Evidence suggest that cases with prior failed CTO-PCI may have significantly lower procedural and technical success rates as compared to those undergoing first-attempt CTO-PCI. There may be no difference in the risk of complications between the two groups. High heterogeneity precludes strong conclusions. Further studies are needed to improve the quality of evidence.

***Registration No:*** PROSPERO under the number CRD420251046077.

## INTRODUCTION

Chronic total occlusions (CTO) are among the most difficult lesions to treat with percutaneous coronary intervention (PCI). Despite a significant frequency of CTO (18-52% among patients having coronary angiography), these lesions were rarely revascularized.^1^ Historically, PCI for CTO was associated with substantial complications and low success rates.^2^ However, with continual advancements in CTO equipment and technology, clinical results have significantly improved over the last decade.^3^ A multicentric randomized controlled trial with 834 patients has shown that CTO-PCI is certainly viable and there is no difference in major adverse cardiovascular events (MACE) between CTO and non-CTO-PCI.^4^ Nevertheless, the procedure remains technically challenging and has a variable success rate ranging from 70-85%, depending upon the clinician’s experience.^5^,^6^ Consequently, the number of patients who have experienced a failed CTO-PCI is substantial in clinical practice and should not be disregarded.

The Japanese-CTO (J-CTO) score remains the most commonly used method for estimating a CTO’s procedural complexity. One of the five factors used by J-CTO to calculate the procedural difficulty of a CTO-PCI is a previous CTO-PCI failure.^7^ Indeed, several patients with unsuccessful CTO-PCI attempts are treated medically rather than re-attempting the procedure.^8^ Presently, there is limited information available in the literature about this challenging cohort. In recent years, a few studies^9^-^11^ have explored the difference in patient outcomes between prior failed and first-attempt CTO-PCI, but no high-quality pooled evidence exists in the literature. We, therefore, conducted the first systematic review and meta-analysis to assess if prior failed CTO-PCI leads to worse outcomes as compared to first-attempt CTO-PCI.

## METHODOLOGY

We followed the Preferred Reporting Items for Systematic Reviews and Meta-Analysis (PRISMA) statement for the review.^12^ Meta-analysis protocol was registered on PROSPERO under the number CRD420251046077.

### Eligibility criteria:

Inclusion in this meta-analysis was restricted to studies meeting the following criteria:

- The study population was CTO patients undergoing PCI.
- Study group consisting of patients with prior failed PCI for CTO.
- Comparative group consisting of patients with first-attempt PCI for CTO.
- The study design was cohort, case-control, or cross-sectional.
- Outcomes reported were procedural or technical success and complications.
- We excluded studies:
- Without a control group.
- Not reporting any of the outcomes of interest.
- where separate data for the study and control group could not be extracted.
- Reporting on the same dataset with overlapping study periods.
- Published only as conference abstracts.

### Search and selection of studies:

Two independent reviewers (CL & WS) conducted a thorough search of the PubMed, Embase, Scopus, and Web of Science literature databases. Studies published between the establishment of these databases and May 15th, 2025 were eligible. No language restrictions were imposed. Keywords used were: chronic total occlusion, CTO, total coronary occlusion, coronary artery total occlusion, percutaneous coronary intervention, PCI, coronary angioplasty, prior failed, previously failed, previously unsuccessful, failed attempts, previous failure, and reattempt. We carefully checked the references of all included articles to identify additional investigations. A search was also run on Google Scholar to identify studies in gray literature.

We eliminated duplicate research after importing the database-searched articles into EndNote version X9 (Thomson Reuters, New York, NY, USA). The same two evaluators subsequently conducted an independent screening of the studies to ascertain their inclusion in their review. This was accomplished by performing a thorough review of the article titles and abstracts. Relevant studies identified by either reviewer then underwent rigorous text analysis before being included. Discussion with a third reviewer (AW) ensured that any disagreements that arose between the two reviewers were eventually resolved.

### Data extraction:

Data extraction was carried out by two reviewers (CL & WS) and cross-checked by the third reviewer (AW). Information obtained from the studies included multiple aspects of the article encompassing publication details like author, location and study design, sample size, demographic details, comorbidities, prior history of myocardial infarction (MI) or coronary artery bypass grafting (CABG), left ventricular ejection fraction (LVEF), prior medications, vessel characteristics, and technical considerations. Details can be noted in Table-I.

**Table-I:** Details of included studies.

	Zheng 2024		Rempakos 2023		Guelker 2021		Sekiguchi 2019		Tanabe 2017		Karacsonyi 2016	
Country	China		Multinational		Germany		Japan		Japan		USA	
Groups	Prior failed	Initial attempt	Prior failed	Initial attempt	Prior failed	Initial attempt	Prior failed	Initial attempt	Prior failed	Initial attempt	Prior failed	Initial attempt
Sample size	49	147	1852	7541	253	366	820	3233	59	251	215	1017
Age (years)	60	64	63.6	64.5	61	62	65.3	67.2	70.2	69.8	64.4	65.6
Males (%)	81.6	84.4	82.8	80.8	80.6	83.6	87.6	85.2	83.1	86.1	81.4	85.5
Current smoker (%)	42.9	44.2	24.2	26.7	47.8	44.8	54.3	54.4	33.9	41	21	30
Diabetics (%)	38.8	44.2	41.2	43	24.1	27.3	45.9	44.9	57.6	53	38.8	45.3
Hypertensive (%)	67.3	68	89	89.4	81.4	81.4	79.4	76.2	83.1	80.9	89	90
Dyslipidemia (%)	71.4	68.7	87.9	84.3	NR	NR	83.8	76.2	69.5	70.9	95.2	94.7
Prior MI (%)	NR	NR	46.9	44.9	37.5	30.9	NR	NR	52.5	43.8	44	42
Prior CABG (%)	NR	NR	27.3	28.2	9.1	9.6	6.7	7.8	28.8	20.7	31	35
LVEF (%)	57.9± 10.5	56.4± 12.2	51.2± 12.4	50.3± 13	NR	NR	56± 12.3	54.6± 13	53.2± 13	54± 15.7	53± 13	50± 14
Heart failure (%)	20.4	15.6	25.1	29.5	NR	NR	NR	NR	28.8	30.7	19	30
Aspirin (%)	96.8	99.3	NR	NR	NR	NR	NR	NR	NR	NR	NR	NR
Statin (%)	96.8	99.3	NR	NR	NR	NR	NR	NR	NR	NR	NR	NR
Stable angina (%)	28.6	29.9	73.3	65.8	NR	NR	NR	NR	NR	NR	NR	NR
Multivessel disease (%)	87.8	89.2	NR	NR	67.9	79	43.6	55.1	NR	NR	NR	NR
***Target vessel***												
LAD (%)	51	51	24.5	26.7	20.6	32.5	30.6	32.2	37.3	20.7	22	23
LCX (%)	8.2	4.8	18.2	19.1	14.2	10.9	11.6	18.9	15.3	36.3	19	19
RCA (%)	40.8	44.2	55.2	52	65.2	54.9	57.2	48.6	44.1	36.7	58	58
Syntax score	19.5	23.3	NR	NR	NR	NR	14.9	17.2	33	30.6	NR	NR
J-CTO score	≥2: 77.5	≥2: 38.8	3.33± 1.16	2.12± 1.19	>3: 62.5	>3: 18.6	2.86± 1.03	1.68± 1.05	3.31± 1	1.43± 1.1	2.4± 1.13	3.28± 1.29
In-stent occlusion (%)	NR	NR	22.1	15	6.3	18.2	11.2	13.2	18.6	19.5	10.5	28.4
Ostial (%)	NR	NR	NR	NR	8.7	14.2	NR	NR	33.9	30.7	NR	NR
Blunt stump (%)	NR	NR	55.3	50.6	58.2	70	22.4	21.6	57.6	48.6	64	58
Calcification (%)	NR	NR	51.5	43.4	65.6	77.5	55.7	50.2	52.5	24.7	56.6	57.4
Tortuous vessel (%)	NR	NR	36.4	26.5	NR	NR	28.2	21.5	45.8	23.9	34.5	34.8
Femoral access (%)	49	27.9	NR	NR	NR	NR	NR	NR	89.8	72.1	NR	NR
Retrograde approach (%)	32.7	3.4	36.4	28.2	32.4	12	NR	NR	55.9	13.9	27	26
IVUS (%)	22.4	19	NR	NR	NR	NR	NR	NR	79.7	80.9	NR	NR
Contrast volume (mL)	319.5	258.6	205	200	200	200	241.5	216.4	203.9	189.4	260	260
Fluoroscopy time (min)	66.8	46.7	46.9	40.4	37	28	NR	NR	81.1	52.2	45	55
Stent implantation (%)	84.6	95.9	100	100	100	100	NR	NR	100	100	NR	NR
NOS score	9		7		7		7		7		7	

LAD, left anterior descending artery; LCX, left circumflex; LVEF, left ventricular ejection fraction; RCA, right coronary artery; CTO, chronic total occlusion; J-CTO, Japanese CTO; IVUS, intravascular ultrasound; MI, myocardial infarction; CABG, coronary artery bypass grafting; NR, not reported; NOS, Newcastle Ottawa scale

Significant differences between groups highlighted with bold.

The endpoints of the study were procedural & technical success and complications. All definitions reported by the studies were acceptable. All complications reported by the studies were extracted and a meta-analysis was conducted if at least two studies reported the same complication.

### Risk of bias analysis:

Each article was given a quality score between zero and nine by the two researchers (CL & AW) using the Newcastle Ottawa Scale (NOS).^13^ The evaluation was carried out in three areas: participant selection, group comparability after confounding correction, and exposure or outcome identification. Each of the three categories has a maximum possible point total of four, two, and three, respectively. The third author (WS) was consulted to settle any disagreements.

### Data analysis:

We pooled data on baseline characteristics as well as review outcomes in the DerSimonian and Laird random-effects meta-analysis model. Odds ratio (OR) with 95% confidence intervals (CI) were calculated for dichotomous data. Mean difference (MD) was generated for continuous variables. Statistical significance was considered for p-values less than 0.05, which was the threshold used for all analyses. Data analysis was conducted in “Comprehensive Meta-analysis” (Version 3) software. Heterogeneity among studies was assessed through Cochran’s Q statistic and the I^2^ index. I^2^ of over 50% and/or P < 0.05 indicated significant heterogeneity. Sensitivity analysis was conducted for success rates. Certainty of evidence was examined by GRADE.

## RESULTS

**Fig.1:**
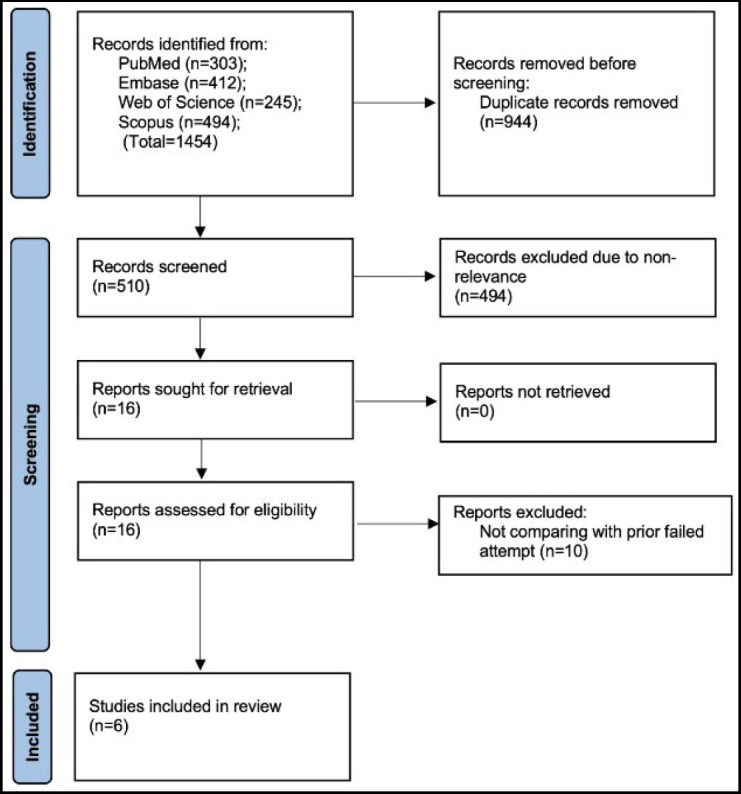
Study flow chart.

Initially, 1,454 publications were discovered from PubMed (303), Embase (412), Scopus (494), and Web of Science (245). After duplicate citations were removed, 510 original papers were subjected to preliminary screening. After reviewing the titles and abstracts, 494 papers were discarded, leaving only 16 for the full-text review. Ten studies were eliminated as they were non-comparative. Six studies^9^-^11^,^14^-^16^ were included in the meta-analysis (Fig.1). No disagreements were noted among the reviewers for the selection of any study. No additional study was found in the gray literature.

### Baseline characteristics:

All studies were retrospective analyses of prospective registries (Table-I). The total sample size of the studies was 15803 of which 3248 (20.6%) patients had prior failed PCI for CTO. All studies predominantly included elderly patients.

We conducted a meta-analysis of baseline differences between the study and control groups. Results are displayed in Table-II. The mean age of patients in the prior failure group was significantly lower. However, there were no significant differences in baseline patient characteristics, namely, male gender, current smokers, diabetes mellitus, hypertension, dyslipidemia, prior MI, prior CABG, LVEF, heart failure, and stable angina at presentation. In terms of disease characteristics, there was no statistically significant difference in multivessel disease, target vessel, in-stent occlusion, ostial CTO, blunt stump, and calcifications between the two groups. However, the J-CTO score of the prior failure group was significantly higher than the first attempt group. The prior failure group also had a significantly higher incidence of tortuous vessels. In terms of procedural characteristics, femoral access and retrograde approach were more frequently used in the prior failure group.

**Table-II:** Meta-analysis of difference in baseline characteristics of patients in the included studies.

Variable	Studies	Effect size	I^2^
Age	6	MD: -1.25 95% CI: -2.15, -0.35	15
Males	6	OR: 0.96 95% CI: 0.74, 1.25	39
Current smoker	6	OR: 0.88 95% CI: 0.69, 1.13	41
Diabetics	6	OR: 0.92 95% CI: 0.72, 1.18	0
Hypertensive	6	OR: 1.03 95% CI: 0.879, 1.34	0
Dyslipidemia	5	OR: 1.3 95% CI: 0.97, 1.75	8
Prior MI	4	OR: 1.19 95% CI: 0.88, 1.61	0
Prior CABG	5	OR: 0.96 95% CI: 0.73, 1.26	0
LVEF	4	MD: 1.04 95% CI: -0.04, 2.13	0
Heart failure	4	OR: 0.78 95% CI: 0.56, 1.09	48
Stable angina	2	OR: 1.28 95% CI: 0.83, 1.99	24
Multivessel disease	3	OR: 1.19 95% CI: 0.88, 1.61	0
***Target vessel***			
LAD	6	OR: 0.93 95% CI: 0.72, 1.20	70
LCX	6	OR: 0.83 95% CI: 0.63, 1.08	81
RCA	6	OR: 1.22 95% CI: 0.96, 1.56	50
J-CTO score	4	MD: 0.84 95% CI: 0.13, 1.56	99
In-stent occlusion	5	OR: 0.75 95% CI: 0.56, 0.99	95
Ostial	2	OR: 0.80 95% CI: 0.47, 1.35	66
Blunt stump	5	OR: 1.06 95% CI: 0.82, 1.37	77
Calcification	5	OR: 1.18 95% CI: 0.91, 1.53	89
Tortuous vessel	3	OR: 1.68 95% CI: 1.21, 2.34	53
Femoral access	2	OR: 2.80 95% CI: 1.48, 5.37	0
Contrast volume (mL)	6	MD: 11.6 95% CI: 7.50, 15.81	85
Fluoroscopy time (min)	5	MD: 6.32 95% CI: 4.70, 7.95	85
Retrograde approach	5	OR: 3.12 95% CI: 1.62, 5.98	94

OR, odds ratio; MD, mean difference; CI, confidence intervals; LAD, left anterior descending artery; LCX, left circumflex; RCA, right coronary artery; J-CTO, Japanese chronic total occlusion; MI, myocardial infarction; CABG, coronary artery bypass grafting; LVEF, left ventricular ejection fraction. Significant differences highlighted in bold.

### Risk of bias:

The risk of bias analysis based on reviewers’ judgment is presented in Table-I. Only one study^16^ used propensity score matching to take into account baseline differences between the study populations. Except for this study,^16^ no other study scored points for the comparability of groups. Five studies received a score of seven, indicating medium risk of bias, and one study received a score of nine, indicating low risk of bias.

### Outcomes:

**Fig.2:**
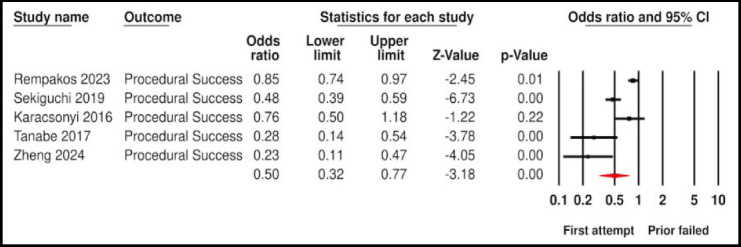
Meta-analysis of procedural success between prior failed and first attempt CTO-PCI groups.

**Fig.3:**
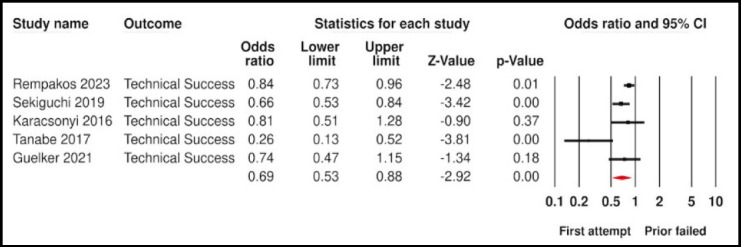
Meta-analysis of technical success between prior failed and first attempt CTO-PCI groups.

Five studies reported data on procedural success and five reported data on technical success. In general, technical success was defined as CTO revascularization with achievement of <30% or 50% residual diameter stenosis within the treated segment and restoration of TIMI Grade-3 anterograde flow. Procedural success was defined as technical success without MACE. Meta-analysis showed that procedural (OR: 0.50 95% CI: 0.32, 0.77 I^2^=89%) and technical success (OR: 0.69 95% CI: 0.53, 0.88 I^2^=68%) was significantly lower in cases with prior failed CTO-PCI as compared to those undergoing first attempt CTO-PCI (Fig.2 & 3). On sensitivity analysis, the results of procedural and technical success were stable on the exclusion of all studies. Certainty of evidence was very low on GRADE (Supplementary Table II).

**Supplementary Table-I:** Search strategy.

PubMed	(((((chronic total occlusion) OR (CTO)) OR (Total coronary occlusion)) OR (Coronary artery total occlusion)) AND (((percutaneous coronary intervention) OR (PCI)) OR (coronary angioplasty))) AND ((((((prior failed) OR (previously failed)) OR (previously unsuccessful)) OR (failed attempts)) OR (previous failure)) OR (reattempt))
Embase	1. ‘chronic total occlusion’ OR ‘CTO’ OR ‘Total coronary occlusion’ OR ‘Coronary artery total occlusion’
	2. ‘percutaneous coronary intervention’/exp ‘percutaneous coronary intervention’ OR ‘PCI’ OR ‘coronary angioplasty’
	3. ‘prior failed’ OR ‘previously failed’ OR ‘previously unsuccessful’ OR ‘failed attempts’ OR ‘previous failure’ OR ‘reattempt’
	4. #1 AND #2 AND #3
Scopus	(TITLE-ABS-KEY-AUTH ((chronic total occlusion) OR (CTO) OR (Total coronary occlusion) OR (Coronary artery total occlusion)) AND (TITLE-ABS-KEY-AUTH(percutaneous coronary intervention) OR (PCI) OR (coronary angioplasty)) AND (TITLE-ABS-KEY-AUTH(prior failed) OR (previously failed) OR (previously unsuccessful) OR (failed attempts) OR (previous failure) OR (reattempt))
Web of Science	(((((chronic total occlusion) OR (CTO)) OR (Total coronary occlusion)) OR (Coronary artery total occlusion)) AND (((percutaneous coronary intervention) OR (PCI)) OR (coronary angioplasty))) AND ((((((prior failed) OR (previously failed)) OR (previously unsuccessful)) OR (failed attempts)) OR (previous failure)) OR (reattempt))

**Supplementary Table-II:** GRADE assessment of evidence.

	Procedural success	Technical success
Number of studies	5	5
***Downgrade quality of evidence***		
Risk of bias	Very serious*	Very serious*
Inconsistency	No	No
Indirectness	No	No
Imprecision	No	No
***Publication bias***		
***Upgrade quality of evidence***		
Large effect	No	No
Plausible confounding	No	No
Dose-response	No	No
Overall certainty of Evidence	Very low	Very low

* Only one included study used propensity score matching; most studies did not adequately match or adjust for baseline differences between groups.

**Fig.4:**
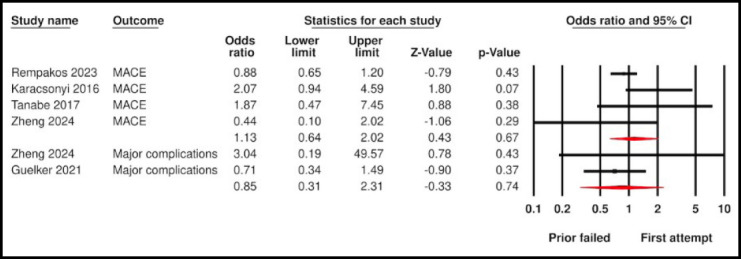
Meta-analysis of MACE and major complications between prior failed and first attempt CTO-PCI groups.

**Fig.5:**
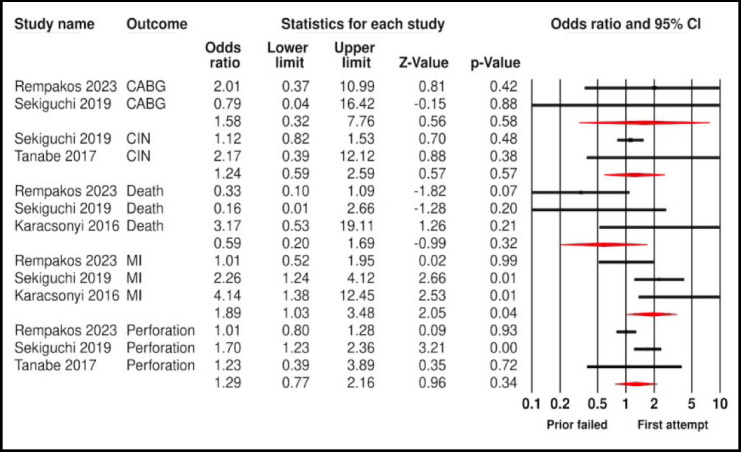
Meta-analysis of emergency CABG, CIN, death, MI, and perforation between prior failed and first attempt CTO-PCI groups.

**Fig.6:**
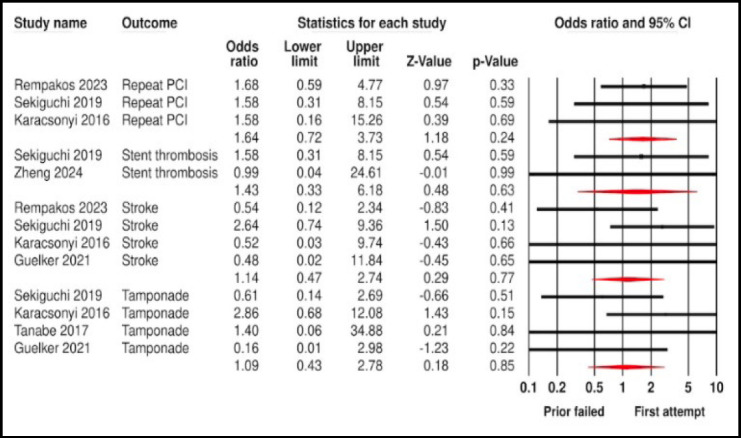
Meta-analysis of repeat PCI, stent thrombosis, stroke, and cardiac tamponade between prior failed and first attempt CTO-PCI groups.

The meta-analysis did not demonstrate a statistically significant difference in the risk of MACE (OR: 1.13 95% CI: 0.64, 2.02 I^2^=47%) and Major complications (OR: 0.85 95% CI: 0.31, 2.31 I^2^=0%) between the two groups (Fig.4). On detailed analysis of individual complications, we noted no significant difference in the risk of emergency CABG (OR: 1.58, 95% CI: 0.32, 7.76, I^2^=0%), contrast-induced nephropathy (CIN) (OR: 1.24, 95% CI: 0.59, 2.59, I^2^=0%), death (OR: 0.59, 95% CI: 0.20, 1.69, I^2^=61%), or perforation (OR: 1.29, 95% CI: 0.77, 2.16, I^2^=69%) between prior failed and first-attempt CTO-PCI groups. However, MI was more frequently observed in the prior failed CTO-PCI group (OR: 1.89, 95% CI: 1.03, 3.48, I^2^=65%, P=0.04), although this finding should be interpreted cautiously because of the limited number of studies and substantial heterogeneity(Fig.5). The pooled analysis also did not demonstrate a significant difference between repeat PCI ((OR: 1.64, 95% CI: 0.72, 3.73, I^2^=0%), stent thrombosis (OR: 1.43, 95% CI: 0.33, 6.18, I^2^=0%), stroke (OR: 1.14, 95% CI: 0.47, 2.74, I^2^=7%), or cardiac tamponade (OR: 1.09, 95% CI: 0.43, 2.78, I^2^=25%)) between the two groups (Fig.6).

## DISCUSSION

This is the first-ever review in the literature that aimed to examine the impact of prior failed attempts on subsequent CTO-PCI results. The key findings from our meta-analysis are as follows:

- Patients with previous failure have lower age, higher Japanese-CTO score, higher incidence of tortuous vessels, required increased contrast volume, and had higher fluoroscopy time.
- The retrograde approach was more frequently used in prior failure cases.
- Cases with prior failure had significantly lower odds of both technical and procedural success rates as compared to those undergoing first-attempt CTO-PCI.
- There seems to be no difference in the risk of complications between the two groups.

At present, PCI for CTO is advised for symptom alleviation when pharmacological treatment is ineffective. Numerous studies have proved the beneficial effects of CTO-PCI on alleviating angina and enhancing quality of life.^17^,^18^ However, most randomized controlled trials have compared outcomes of PCI vs medical therapy and these trials frequently exclude patients with severe disease and high symptom load, the one most likely to benefit from CTO PCI.^4^,^19^ Another drawback of these trials is they don’t stratify outcomes based on the success of the procedure, which has a significant impact on subsequent outcomes. A meta-analysis of contemporary studies has shown that while success rates of CTO-PCI are high in current practice, about 14-19% of patients still experience failure. MACE and all-cause mortality were significantly reduced in successful cases while failure cases continued to experience worse outcomes.^20^ Management of failure cases is either by medical therapy or by the more traumatic CABG, with only about 10% of cases receiving re-attempt PCI.^21^ There remains limited literature on re-attempted CTO-PCI and single-arm studies suggest that the success of re-attempt CTO-PCI varies from 68 to 81%.^21^,^22^ Research also shows that successful re-attempted CTO-PCI leads to a significant reduction of MACE on long-term follow-up.^22^ Moreover, despite the re-attempted cases having high J-CTO scores, complication rates remain low in experienced hands especially in high-volume CTO centers.^21^

In our meta-analysis of six comparative studies with a large cohort, we have partly reinforced the results of such single-arm studies. The analysis showed that technical (84.8% vs 88%) and procedural success (81.5% vs 86.3%) of re-attempted CTO-PCI was significantly lower than first-attempt cases. The results did not change on sequential exclusion of studies indicating consistency of outcomes. On the other hand, the risk of complications did not differ between the two groups. However, we must advise caution in the interpretation of the results as very few studies were included in specific meta-analyses of individual complications.

The lower success rates in the prior failed group could be attributed to the baseline differences in lesion characteristics. Prior failure was associated with a difficult reattempt at CTO recanalization, evidenced by an elevated J-CTO score and increased tortuosity of vessels in this subgroup. This may have led to higher contrast volumes and increased fluoroscopy time, indirectly reflecting the difficulty of the procedure. In our meta-analysis, patients with a history of unsuccessful CTO-PCI attempts had a 0.84 point higher J-CTO scores compared to those who underwent initial CTO-PCI attempts. The J-CTO score, a commonly utilized scoring system, is often employed to estimate the likelihood of effective guidewire crossing within 30 minutes, indicating the complexity of percutaneous recanalization of CTO lesions. Prior failed attempts are worth one point in the scoring system, with a J-CTO score of ≥2 indicating a more challenging lesion.^7^ In this context, research has suggested that intravascular ultrasound can be used to enhance outcomes. Intravascular ultrasonography aids in identifying the actual lumen, facilitating wire crossing, and optimizing the PCI process, particularly in severely calcified CTO segments.^23^ Secondly, specialized CTO devices, such as the CrossBoss and the Stingray catheter, can also be used to enhance success rates in CTO-PCI procedures.^24^,^25^ In the present review, we could not examine the impact of these advanced technologies on success rates due to a lack of adequate data.

Several other factors can influence the outcomes of re-attempted CTO-PCI. Zhong et al.^26^ have shown that subintimal plaque modification utilizing guidewire crossing, treatment with high-volume operators, a bidirectional technique, and a reattempt interval of fewer than 90 days enhances the technical success of re-attempt PCI. The investment procedure is recommended by the global CTO crossing algorithm if CTO crossing fails.^27^ Although research indicates that subintimal plaque modification with guidewire crossing had higher success rates in subsequent reattempt procedures,^26^ some studies do not demonstrate a correlation between the performance of an investment procedure and procedural success after reattempting CTO PCI.^15^ Secondly, CTO-PCI is a highly technique-sensitive procedure with success rates directly proportional to operator volume.^28^ Likewise, success rates of re-attempt CTO-PCI are also highly dependent on operator experience given the increased complexity of the lesions. This has been delineated by Rempakos et al.^15^ who noted technical success rates of 87.4% with high-volume operators and only 80.8% with low-volume operators in reattempt cases.

We noted that the retrograde approach was more frequently utilized in the re-attempt group. Research shows that the efficacy of CTO PCI is significantly enhanced by the retrograde approach, particularly in highly complex CTOs.^29^ The global CTO crossing algorithm also recommends a retrograde procedure for patients with proximal cap uncertainty, poor distal vascular quality, or if anterograde crossing fails.^27^ However, the retrograde technique has greater complication rates and should only be performed by skilled physicians.^29^

### Limitations:

Only six studies could be included and all were registry-based studies with high possibilities of data-entry errors and selection bias. Secondly, we did not find adequate data on the time interval between prior failure and reattempt PCI in the study group. It is suggested that timing between initial procedure and reattempt can influence patient outcomes and success rates are higher when repeat intervention is performed within two months.^30^ Thirdly, most of the meta-analysis on specific complications had a limited number of studies which reduces the statistical power and confidence in the outcomes. Fourthly, several operators were involved in treating patients in the included studies. The impact of operator experience could not be thoroughly assessed in this review. Lastly, the baseline characteristics were not exactly matched between the two groups. The lower success rates in the prior failed group could be due to the baseline differences in lesion characteristics. The data analysis was also based on crude data and not adjusted data. Hence, the results must be interpreted with caution.

The study has important clinical implications. While re-attempted CTO-PCI had lower procedural success, the similar complication risk indicates that repeat intervention can be considered in carefully selected patients. Re-attempt CTO-PCI may be required in those with persistent angina or significant ischemic burden despite optimal medical therapy. Use of advanced imaging, use of dedicated CTO devices, or adoption of alternative strategies like the retrograde approach may be helpful in re-attempt cases. However, it should be performed at high-volume centers with experienced operators. Further, high quality propensity score matched studies taking into account baseline variables are needed to improve the quality of evidence.

## CONCLUSIONS

Patients with previous unsuccessful CTO-PCI may have diminished procedural and technical success rates relative to those undergoing CTO-PCI for the first time. The risk of complications may be identical between the two patient groups. High heterogeneity precludes strong conclusions. Additional research is required to enhance the quality of evidence.

### Authors’ contributions:

**WS and AW:** Literature search, study design and manuscript writing.

**WS, AW and CL:** Data collection, data analysis and interpretation. Critical review.

**WS and AW:** Manuscript revision and validation and is responsible for the integrity of the study.

All authors have read and approved the final manuscript.
